# The Acetone Extract of *Sclerocarya birrea* (Anacardiaceae) Possesses Antiproliferative and Apoptotic Potential against Human Breast Cancer Cell Lines (MCF-7)

**DOI:** 10.1155/2013/956206

**Published:** 2013-03-20

**Authors:** Nicoline Fri Tanih, Roland Ndip Ndip

**Affiliations:** ^1^Department of Biochemistry and Microbiology, Faculty of Science and Agriculture, University of Fort Hare, P/Bag X1314, Alice 5700, South Africa; ^2^Department of Microbiology and Parasitology, Faculty of Science, University of Buea, P.O. Box 63, Buea, Cameroon

## Abstract

Interesting antimicrobial data from the stem bark of *Sclerocarya birrea*, which support its use in traditional medicine for the treatment of many diseases, have been delineated. The current study was aimed to further study some pharmacological and toxicological properties of the plant to scientifically justify its use. Anticancer activity of water and acetone extracts of *S. birrea* was evaluated on three different cell lines, HT-29, HeLa, and MCF-7 using the cell titre blue viability assay in 96-well plates. Apoptosis was evaluated using the acridine orange and propidium iodide staining method, while morphological structure of treated cells was examined using SEM. The acetone extract exhibited remarkable antiproliferative activities on MCF-7 cell lines at dose- and time-dependent manners (24 h and 48 h of incubation). The extract also exerted apoptotic programmed cell death in MCF-7 cells with significant effect on the DNA. Morphological examination also displayed apoptotic characteristics in the treated cells, including clumping, condensation, and culminating to budding of the cells to produce membrane-bound fragmentation, as well as formation of apoptotic bodies. The acetone extract of *S. birrea* possesses antiproliferative and apoptotic potential against MCF-7-treated cells and could be further exploited as a potential lead in anticancer therapy.

## 1. Introduction

Cancer diseases are the second largest single cause of death claiming over six million lives every year following closely behind cardiovascular diseases in developed countries and the third after infectious and cardiovascular diseases in developing countries. They are distinguished by an abnormal proliferation of cells [1, 2]. Diagnosis resulting to cancer detection is very high in this century with higher prevalence rates of breast, colon, and cervical cancers [3, 4]. Cervical and breast cancers appear to represent two common forms of cancer in women [4]. Breast cancer accounts for approximately 30% of all cancer diagnosed in women in the United States [4, 5] and is the second leading cause of cancer death in women. Cervical cancer on the other hand remains globally an important cause of female mortality [6] with a high prevalence in sub-Saharan Africa [7]. 

Continuous effort to establish a better anticancer agent provides some hope to mankind. In generation past, prior to the advent of modern allopathic medicine, herbs and substances derived from plants have been the mainstay of traditional medicine around the world [8]. There has been a recent upsurge in the use of natural products in the management of diseases. Research on plants used in various types of ethnic medicine has led to the discovery of many valuable drugs, including taxol, camptothecin, vincristine, and vinblastine [9, 10] which are used in the treatment of cancer.

*Sclerocarya birrea* constitutes one of the most highly valued indigenous trees of Southern Africa [11] and frequently used plant species. It belongs to the family Anacardiaceae and commonly known as “cider tree” or “marula” (in English), “maroela” (in Afrikaans), or “umganu” (in Zulu) [11]. It is a medium size to large deciduous tree with a trunk that is erect. This indigenous, drought-tolerant multipurpose tree is widely distributed in sub-Saharan Africa [12]. The potency of this plant in sustainable health management is unquestionable. The stem-bark, roots, and leaves have been used in South Africa and in some other African countries for the management of an array of human ailments, including malaria, dysentery, headaches, toothache, backache and body pains, infertility, schistosomiasis, epilepsy, and diabetes mellitus [12, 13]. 

Pharmacological studies by various groups of investigators have shown that *S. birrea* possesses antidiarrhoeal, antidiabetic, antiinflammatory, antimicrobial, antiplasmodial, antihypertensive, anticonvulsant, and antinociceptive properties, thus lending pharmacological support to the plant's folkloric, ethnotherapeutic uses in South African traditional medicine [11]. The plant has a high polyphenolic content and good antioxidant property [13, 14]. 

Various investigators have conducted studies on different plants employing both *in vivo* and *in vitro* approaches to evaluate their anticancer potential following an intensive quest for anticancer agents from plants and other natural sources [15–18]. Research on *S. birrea* has been extensive but largely on their antimicrobial, antiprotozoal, and antioxidant activities of which the plant and associated extracts have proven its potential. Studies related to phytochemistry have revealed the presence of varied types of phenolic compounds [11, 13]. Polyphenols have been found useful as antioxidants, antimutagens, scavengers of free radicals and therefore have implications in the prevention of pathologies such as cancer and cardiovascular disease [19]. 

However, we are not aware of any information on the anticancer property of this plant keeping in mind its inherent nutritional and pharmaceutical properties. This study was therefore undertaken to examine the antiproliferative and apoptotic effect of crude extracts (acetone and water extract) of *S. birrea* using different mammalian cell lines in an effort to validate its safety and potential as a possible lead for anticancer drug development. 

## 2. Materials and Methods

### 2.1. Preparation of Plant Material

Plant material was harvested from different trees in the Venda region, North of South Africa and transported to the University of Fort Hare. The plant was identified by botanists at the School of Biological Sciences, University of Fort Hare, Alice and voucher specimens (GEUFH01) deposited at the school's herbarium. The plant part (stem bark) was washed with tap water, chopped into small pieces, and dried at 40°C in a hot air oven (Memment 854, Western Germany) for one week. It was powdered using a blender (ATO MSE mix, England) and further macerated in acetone and water, respectively, and the crude extract was obtained as previously described [20].

### 2.2. Plants Extract Preparation for Screening

Stock solutions containing plant extracts were prepared fresh on the day of the experiment. Dimethyl sulfoxide (DMSO) (Merck Diagnostic, South Africa) was used for dissolving the extracts to make a stock solution at 200 mg/mL. Final test concentrations were obtained by diluting the stock solution with Dulbecco's minimum essential medium (DMEM) supplemented with 10% fetal bovine serum (FBS). The final concentration of DMSO to which cell cultures were exposed never exceeded 0.25%. 

### 2.3. Cell Line Growth and Maintenance

This study was conducted on three cancer cell lines HT-29 (colon cancer), HeLa (cervical cancer), MCF-7 (breast cancer), and a noncancerous cell line (human Chang liver cell). Vials containing cells were taken from liquid nitrogen stocks. The cells were thawed in a water bath (37°C) and transferred to a 25 mm^3^ culture flask (TPP, Switzerland). A 1 mL thawed cell stock was diluted with 9 mL prewarmed DMEM containing 10% FBS. The cells were incubated in a 37°C humidified incubator (Shel-Lab, USA), 5% CO_2_ for multiplication and adherence. Maintenance of cells was achieved by splitting the cells until the desired cell number and confluence were reached.

### 2.4. *In Vitro* Cytotoxicity Assay

The Cell Titre Blue Viability (Promega, USA) assay uses the dark blue indicator dye resazurin to measure the metabolic capacity of cells—an indicator of cell viability. Viable cells retain the ability to reduce resazurin into resorufin (pink), which is highly fluorescent. Nonviable cells rapidly lose metabolic capacity and do not reduce the indicator dye and thus do not generate a fluorescent signal. Following 24 h and 48 h incubation periods after treatment with tested extracts (acetone and water) and curcumin which was employed as a positive control, 40 *μ*L of the cell tire blue reagent was added to the plates. Plates were further incubated for 4 h at 37°C. Fluorescence was read at an excitation wavelength of 620 nm and an emission wavelength of 573 nm using an automatic ELISA microplate reader (BioTek, Tokyo, Japan). The IC_50_ value was determined and data was reported as the average of three replicates. 

### 2.5. Apoptosis Assay

Apoptosis was performed according to the method of Mohan et al. [21]. Briefly, MCF-7 cells were plated at a concentration of 1 × 10^6^ cell/mL and treated with acetone extract of *S. birrea* in a 12-well plate at IC_50_ and IC_50_ × 4 concentrations. Experimental controls (positive control and untreated cells) were employed. Plates were incubated in an atmosphere of 5% CO_2_ at 37°C for 24 h and 48 h. The cells were then centrifuged at 300 ×g for 10 min. The supernatant was discarded and the cells were washed twice with phosphate buffered saline (PBS). Ten microliters of fluorescent dyes containing acridine orange (AO) and propidium iodide (PI) was added into the cellular pellet in equal volumes (10 *μ*g/mL), respectively. Freshly stained cell suspension was dropped into a glass slide and covered with a cover slip. Slides were observed under UV-fluorescence microscope within 30 min before the fluorescent color faded accordingly: (i) viable cells appeared to have green nucleus with intact structure; (ii) early apoptotic cells exhibited a bright-green nucleus showing condensation of chromatin in the nucleus; (iii) dense orange areas of chromatin condensation revealed late apoptosis, and (iv) orange intact nucleus depicted secondary necrosis.

### 2.6. DNA Fragmentation Analysis

DNA fragmentation was analysed by agarose gel electrophoresis as previously described by Wang et al. [22], with slight modifications. MCF-7 cells (1.5 × 10^6^ cells/mL) were exposed to the extract for 24 h and harvested by centrifugation. Cell pellets were resuspended in 200 *μ*L of lysis buffer, and DNA was extracted using a Nucleospin protein and DNA extraction kit (Separations, South Africa). DNA solution was transferred to 1.5% agarose gel and electrophoresis was carried out at 67 V for 3/4 h with TAE (Tris 40 mM, sodium acetate 20 mM, EDTA 1 mM) as running buffer. DNA in the gel was visualized using ethidium bromide (0.5 g/mL) under UV light (Alliance 3).

### 2.7. Scanning Electron Microscopy

The morphological characteristics of the cells were determined by scanning electron microscopy (SEM; JSM −6390LV, Jeol, Japan). MCF-7 cell lines (1.5 × 10^6^ cells/mL) were plated in 6 well plates, treated with different concentrations of acetone extract of *S. birrea, *cultured, and later harvested by trypsinization; the cells were washed with 0.1 M cacodylate buffer, centrifuged 2x at 1000 rpm for 3 mins and seeded on poly-L-lysine-coated coverslips which were further fixed with 2.5% glutaraldehyde in 0.1 M cacodylate buffer. Cells were postfixed in 1% osmium tetroxide in 0.2 M PBS, dehydrated through graded ethanols (30, 50, 70, 85, 95%) and critical point dried in CO_2_. Samples were gold-coated using IB3 Ion coater (Eiko, Japan) and observed.

### 2.8. Statistical Analysis

The results are presented as means ± SEM of three independent experiments. Statistical differences among means were determined by one-way ANOVA, and differences between time intervals (24 h and 48 h) were computed using Mann-Whitney test using statistical package Minitab. Differences were considered significant at *P* < 0.05.

## 3. Results 

**Figure 1:**
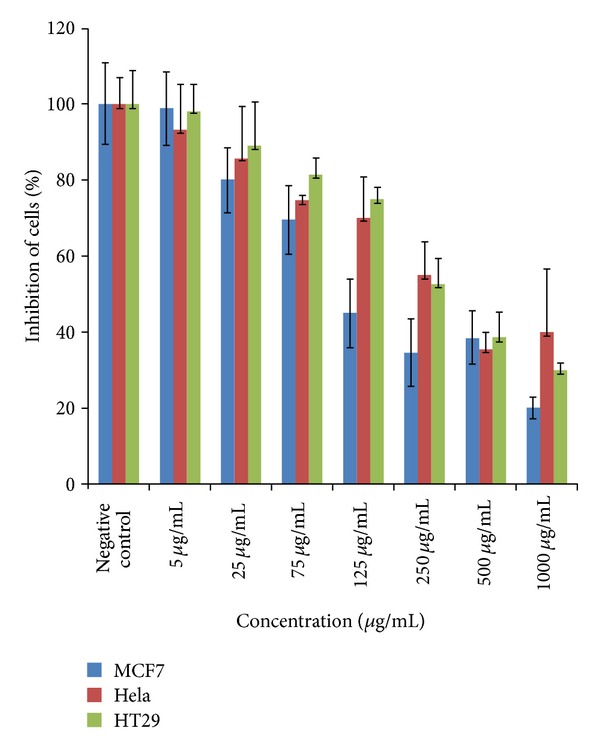
Effect of *S. birrea* acetone extract on proliferation of MCF-7, HT-29 and HeLa cells at 24 h. Each value represented mean ± SEM of six replicates (*n* = 6).

We investigated the anticancer property of acetone and water extracts of the stem bark of *S. birrea* on three different cancer cell lines. Viability assay result showed that the acetone extract exhibited a better antiproliferative effect on the three cell lines (MCF-7, HT-29, and HeLa) compared to the water extract (data not shown). A better antiproliferative activity was observed for the MCF-7 cells being the most inhibited with an IC_50_ = 87.6 *μ*g/mL as opposed to HT-29, and HeLa with values of 686.65 *μ*g/mL and 326.01 *μ*g/mL, respectively (Figure 1).

 The water extract of the plant did not show significant inhibition; hence only the acetone extract was used for further studies. Likewise, since MCF-7 recorded the least IC_50_ value it was subjected to further investigations.

**Figure 2:**
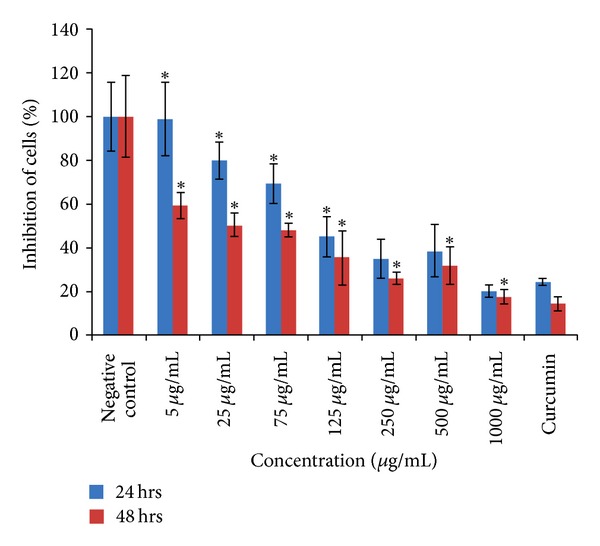
Effect of *S. birrea* acetone extract on proliferation of MCF-7 cells at 24 and 72 h. Each value represented mean ± SEM of six replicates (*n* = 6); *P < 0.05.

The effect of the acetone extract over time (24 h and 48 h) on the MCF-7 cell was investigated. Generally, cells were significantly inhibited in a dose- and time-dependent manners (*P* < 0.05) at both 24 h and 48 h (Figure 2).

 At 24 h of treatment, only at the concentration of 125 *μ*g/mL and above, that the acetone extract significantly inhibited the growth of MCF-7 cells (*P* < 0.05). Meanwhile, after 24 h of incubation, at most tested concentrations (5 *μ*g/mL–1000 *μ*g/mL), the extract exhibited significant inhibitory effects (*P* < 0.05). The inhibitory activities increased and the IC_50_ values decreased as the incubation period was increased from 24 h (87.6 *μ*g/mL) to 48 h (44.71 *μ*g/mL). The activity of curcumin (60 *μ*M) was significantly lower when compared to the tested concentrations of the crude extract. Toxicity of acetone extract of *S. birrea* was tested on human Chang liver cells. Our data suggested that the acetone extract had selective (moderate) toxicity on the cells (data not shown). 

**Figure 3:**
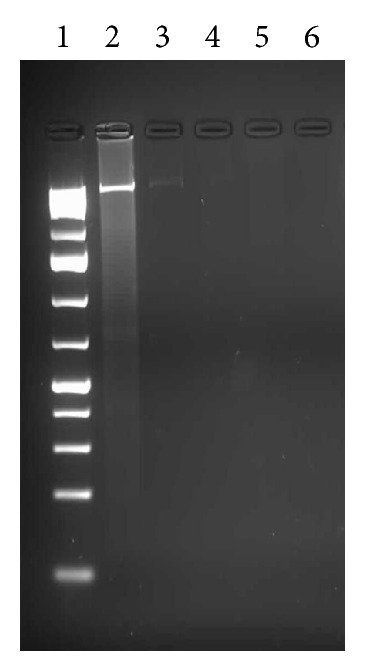
Agarose gel (1,5%) profile of DNA from MCF-7 cells treated with acetone extract of *S. birrea*. Effect of different concentrations of acetone extract (EA) after 24 h incubation. Lane 1: molecular weight marker, lane 2: untreated MCF-7 cells, note that cells were unaffected, lane 3: treated cells with IC_50_ conc. DNA concentration diminishes with a visible faint band. Lane 4: treated cells with IC_50_  ×  2 conc, faint band completely disappears; in subsequent lanes. Lane 5: treated cells with IC_50_  ×  4 conc. Lane 6: treated cells with IC_50_  ×  8 concentration.

In order to investigate whether apoptosis may have played an important role in mediating the cell death of MCF-7, DNA analysis of cells treated with the acetone extract was executed. It was observed that the extract had a significant effect on the DNA. As the concentration of the extract was increased from IC_50_ to IC_50_  ×  4 so was the degradation effect on the DNA (Figure 3). 

Interestingly, we did not notice degradation of DNA in terms of fragments as observed in other studies. However, the mechanism of action of the extract on DNA was not investigated. 

**Figure 4:**
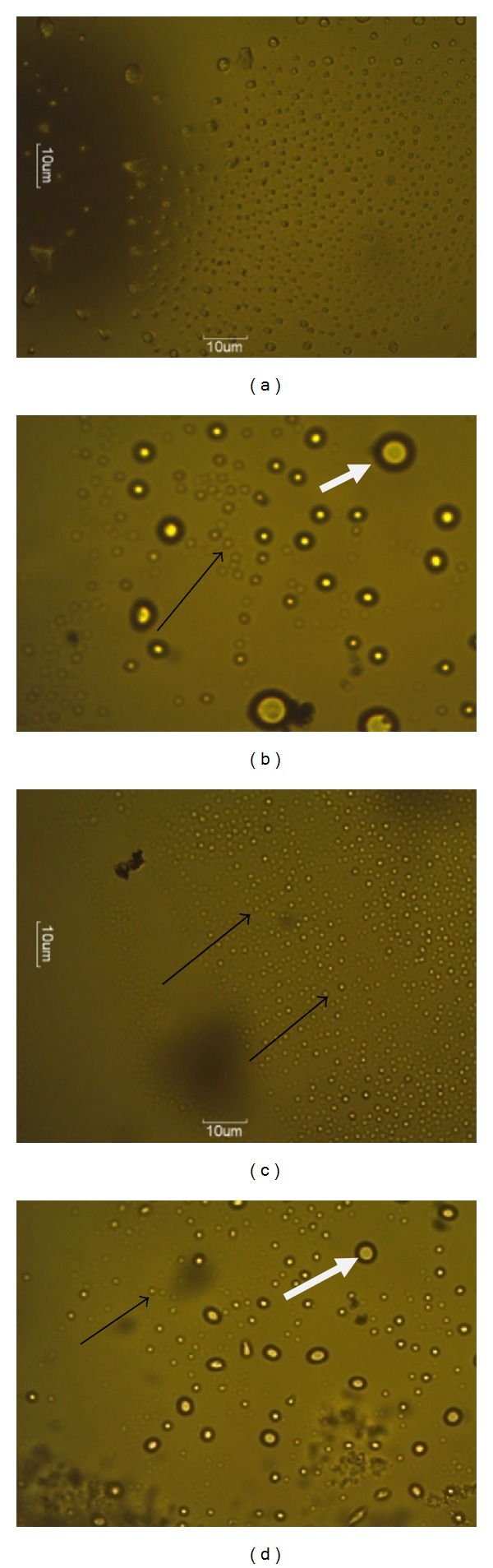
Effect of *S. birrea *acetone extract on MCF-7 cells at 24 h and subjected to acridine orange (Life technology, South Africa) and propidium iodide staining. (a) Negative control of untreated cells depicting viable cells (green nucleus), (b) cells treated with positive control curcumin (60 *μ*M), showing late apoptosis (thick arrow), (c) treated cells with IC_50_ (87.6 *μ*g/mL) concentration indicating early apoptosis (thin arrow), and (d) treated cells with IC_50_  ×  4 showing late apoptosis and secondary necrosis.

 Differential staining and fluorescence microscopy analyses revealed that the plant extract induced apoptosis. Nuclei of untreated negative control cells were not stained in this assay. Treated cells produced orange brown stained nuclei with green nuclei for unaffected cells; similar observation was noted in the positive control cells treated with curcumin (60 *μ*M). The nuclei were specifically stained and evenly distributed. Most of the positive stained nuclei were rounded or oblong in shape as shown in Figures 4(a), 4(b), and 4(d), the extract-treated MCF-7 cells. 

**Figure 5:**
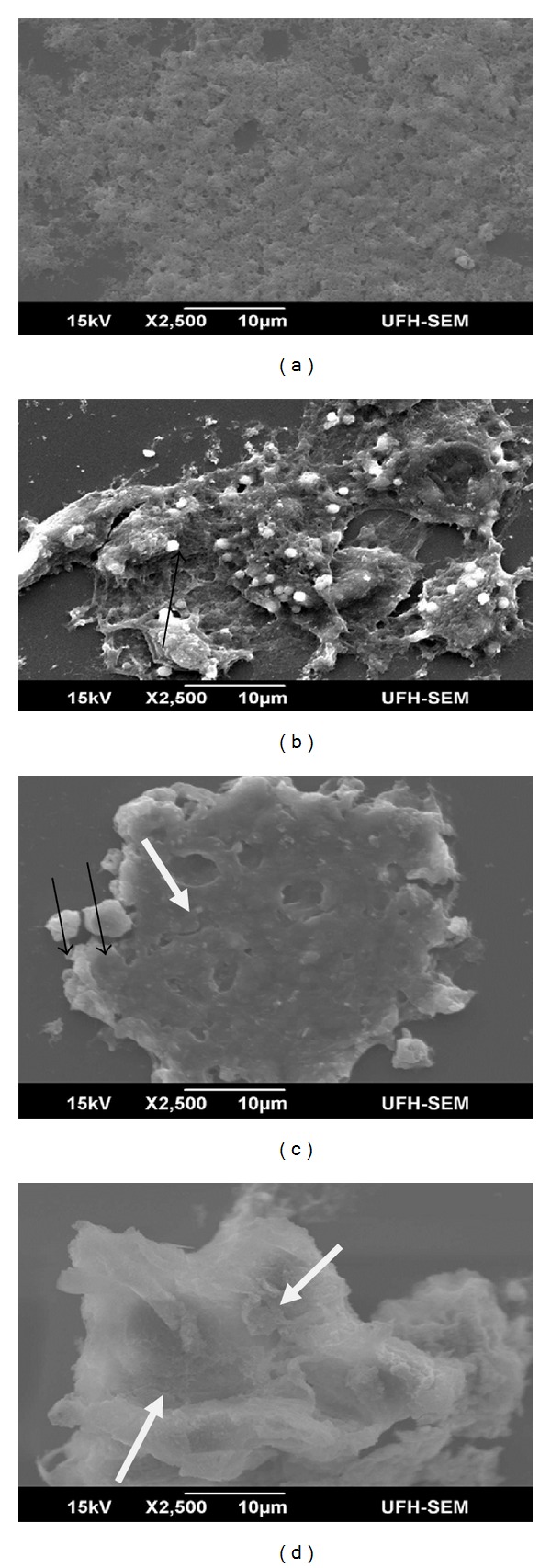
Scanning electron micrographs: (a) untreated MCF7 cells (control), (b) MCF-7 cells treated with curcumin (60 *μ*M) (positive control), note clumping and margination culminating to budding of the cells and formation of apoptotic bodies, (c) treated MCF7 cells in IC_50_ concentration (87.6 *μ*g/mL), (d) treated MCF7 cells in IC_50_  ×  4 concentration note disintegration and blebbing. Apoptotic body (thin black arrow), thick white arrow, cell disintegration.

Scanning electron microscopy examination after treatment with the different extract concentrations (IC_50_ and IC_50_  ×  4) revealed antiproliferation via apoptosis pathway. Morphological modifications were clearly visible. Some of the changes observed include cytoplasm condensation with a pronounced decrease in cell volume, chromatin condensation and fragmentation, plasma membrane blebbing and degeneration of the nucleus into formation of apoptotic bodies (Figures 5(b), 5(c), and 5(d)). Negative control containing untreated cells had cells growing in confluence (Figure 5(a)).

## 4. Discussion

Plant-based research has been paramount in the discovery of biological active compounds, and derivatives from plant have been found with anticancer properties [2, 4, 18, 23]. Previous studies conducted indicated profound activity of the acetone extract of *S. birrea* against different microorganisms including *H. pylori* which has been incriminated in gastric carcinoma [13, 20]. The pharmacological and nutritional benefits of different parts of *S. birrea* have been described both in traditional and modern medicines [11–13]. Against this background and taking into cognisance the dearth of knowledge on its anticancer property, this study aimed at elucidating the *in vitro* anticancer effects of the acetone and water extracts of the stem bark of *S. birrea* as well as its toxicity on human Chang liver cells (normal lines).

Evaluation of viable cells after treatment with the plant extracts was based on the use of the cell titre blue viability assay. Viability assay result indicated that the acetone extract exhibited a better antiproliferative effect on the three cell lines (MCF-7, HT-29, and HeLA) compared to the water extract. This is not surprising particularly because in a previous study by Njume et al. [20] on *H. pylori* isolates, we showed a higher activity with the acetone extract. Also, this lends credence to the fact that screenings of medicinal plants should always include different solvents of the plants, since the variation in secondary compounds between different plants organs can be enormous [11]. 

Inhibition of the cell lines was in a dose- and time-dependent manners with MCF-7 presenting with the least IC_50_ value = 87.6 *μ*g/mL. This result corroborates the findings of other investigators who have demonstrated varying anticancer activity of different plants though some of the plants were more antiproliferative than others [2, 4, 16, 24]. For example, Tan et al. [4] had reported high inhibitory activity of methanol extract of *Pereskia bleo* on breast cancer line with an EC_50_ = 2.0 *μ*g/mL. To the best of our knowledge, our study seems to be the first to report on the anticancer effects of acetone and water extracts of *S. birrea *on these cancer lines. Biomedical literature has indicated the presence of medicinally important chemical constituents in different parts of this plant, notably: polyphenols, tannins, coumarins, flavonoids, triterpenoids, phytosterols, and so forth [11–14, 19]. Furthermore, acetone extract of the stem bark of this plant has been found to possess tannins, flavonoids, with good antifungal and antimicrobial effects [11–14]. Isolation and identification of compounds in the plant extract had revealed high levels of pyrollidine, Terpinen-4-ol, aromadendrene, *α*-gurjunene components of the essential oils of several aromatic plants which exhibits antitumor and anticancer effects [20, 25–27]. Since the extract we used had been characterized in our previous study [27] to contain these compounds, we suggest that the compounds might be responsible for the remarkable antiproliferative activity observed for this extract against the investigated cancer cell lines. 

DNA analysis against the MCF-7 cell lines revealed an apoptotic induced cell death. Sublethal concentrations of the extract (87.6 *μ*g/mL = IC_50_) (Figures 3(c) and 4(d)) induced apoptosis and DNA degradation, however, not in fragments. Our results are contrary to the finding of Leong et al. [24] who reported fragmentation in their DNA using a modified TUNEL assay.

 Stained cells with AO and PI and fluorescence microscopy analysis revealed that the extract induced apoptosis and caused morphological changes of cells. Apoptotic-morphological features have been reported in various studies involving plants and anticancer agents on cancer lines [4, 18, 21]. In our current study, morphological modifications were clearly visible during apoptosis. Some of the changes observed include cytoplasm condensation with a pronounced decrease in cell volume, chromatin condensation and fragmentation, plasma membrane blebbing, and degeneration of the nucleus into membrane bound apoptotic bodies (Figures 4(a), 4(b), and 4(d)) which have been observed in other studies [4, 18, 21]. This is in line with the findings of Tan et al. [4] who reported apoptotic effect with MCF-7 cells treated with methanol extract of *Pereskia bleo*, though a different plant. However, investigations on the effects of this extract on expressions of marker enzymes for apoptosis (p53, caspases, etc.) are needed for verification of this observation. 

## 5. Conclusion

*S. birrea* is frequently used in African traditional medicine, although not for the treatment of cancer; the antiproliferative effects we observed in the current study may complement its use for the treatment and management of cancer in Africa. Our results indicated that the acetone extract inhibited the proliferation of MCF-7 cell line at a dose- and time-dependent manners via an apoptotic programmed cell death. Therefore more studies on its mode of action will be relevant in optimising its potential as a possible lead in anticancer therapy. Both *in vitro* and *in vivo *toxicities should be performed to assess its safety.
